# Clinical guideline SEOM: hepatocellular carcinoma

**DOI:** 10.1007/s12094-015-1451-3

**Published:** 2015-11-25

**Authors:** J. Sastre, R. Díaz-Beveridge, J. García-Foncillas, R. Guardeño, C. López, R. Pazo, N. Rodriguez-Salas, M. Salgado, A. Salud, J. Feliu

**Affiliations:** Medical Oncology Department, Hospital Universitario Clínico San Carlos, Prof. Martín Lagos, s/n, 28040 Madrid, Spain; Medical Oncology Department, Hospital Universitari I Politècnic la Fe, Valencia, Spain; Medical Oncology Department, Hospital Universitario Fundación Jiménez Díaz, Madrid, Spain; Medical Oncology Department, Hospital Universitari de Girona Dr. Josep Trueta, Girona, Spain; Medical Oncology Department, Hospital Universitario Marqués de Valdecilla, Santander, Spain; Medical Oncology Department, Hospital Universitario Miguel Servet, Saragossa, Spain; Medical Oncology Department, Hospital Universitario la Paz, Madrid, Spain; Medical Oncology Department, Complexo Hospitalario de Ourense (CHOU), Madrid, Spain; Medical Oncology Department, Hospital Universitari Arnau de Villanova de Lleida, Lleida, Spain

**Keywords:** Hepatocellular carcinoma, Guidelines, Ablative therapies, Sorafenib

## Abstract

Hepatocellular carcinoma (HCC) represents the second leading cause of cancer-related death worldwide. Surveillance with abdominal ultrasound every 6 months should be offered to patients with a high risk of developing HCC: Child-Pugh A–B cirrhotic patients, all cirrhotic patients on the waiting list for liver transplantation, high-risk HBV chronic hepatitis patients (higher viral load, viral genotype or Asian or African ancestry) and patients with chronic hepatitis C and bridging fibrosis. Accurate diagnosis, staging and functional hepatic reserve are crucial for the optimal therapeutic approach. Characteristic findings on dynamic CT/MR of arterial hyperenhancement with “washout” in the portal venous or delayed phase are highly specific and sensitive for a diagnosis of HCC in patients with previous cirrhosis, but a confirmed histopathologic diagnosis should be done in patients without previous evidence of chronic hepatic disease. BCLC classification is the most common staging system used in Western countries. Surgical procedures, local therapies and systemic treatments should be discussed and planned for each patient by a multidisciplinary team according to the stage, performance status, liver function and comorbidities. Surgical interventions remain as the only curative procedures but both local and systemic approaches may increase survival and should be offered to patients without contraindications.

## Introduction

Hepatocellular carcinoma (HCC) represents the fifth most common cancer in men and the ninth in women (7.5 and 3.4 % of all cancers, respectively). This is the second leading cause of cancer-related death worldwide, with approximately 745,500 deaths during the year 2012. The incidence varies widely according to geographic location so, while in the EU it is approximately 8.6/100,000 people, in certain regions of Asia and Africa this rate reaches up to 120/100,000 people. This is mainly related to the different level of exposure to specific risk factors. HCC frequency is 4–8 times higher in men. The median age for diagnosis is 60 in low-incidence areas. The incidence in Spain is around 17/100,000 for men and 6.5/100,000 for women. With a 9.7/100,000 mortality rate, HCC is the eighth cause of cancer-related death in Spain [1].

HCC is usually diagnosed in cirrhotic patients (60–80 %). Patients with cirrhosis due to chronic hepatitis B virus infection (HBV) have a 100 times increased risk of suffering HCC, thus being the main etiology in high-incidence countries. The risk of HCC in patients with cirrhosis, secondary to the hepatitis C virus (HCV), is 1–2 % per year, causing most of the new cases in Europe. Both the co-HBV infection and alcohol consumption increase the risk. As viral load and active viral replication are associated with a higher likelihood of developing HCC, antiviral therapies can potentially reduce the risk of patients with this kind of chronic hepatitis. Nonalcoholic fatty liver disease (NAFLD) represents an increasingly frequent underlying liver disease in patients with HCC, especially in developed countries [2]. Other causes of chronic hepatitis such as hemochromatosis and aflatoxin are less common etiologies for HCC.

### Surveillance

Surveillance should be offered to patients with a high risk of developing HCC: Child-Pugh A–B cirrhotic patients, all cirrhotic patients on the waiting list for liver transplantation, high-risk HBV chronic hepatitis patients (higher viral load, viral genotype or Asian or African ancestry) and patients with chronic hepatitis C and bridging fibrosis. Despite the increasing incidence of nonalcoholic fatty liver disease in developed countries, surveillance of these patients, although endorsed by some guidelines [3], remains, at the present time, controversial.

An abdominal ultrasound (US) every 6 months is the method of choice, as it has shown to be superior compared to three- and twelve-monthly intervals [4, 5]. There is no role for AFP or other oncomarkers in HCC screening [6]. There are no data to support the use of multidetector computed tomography (CT), or dynamic magnetic resonance imaging (MRI) for surveillance. Appropriate recall procedures should be in place in case a nodule is found in a screening US (new nodules that measure more than 1 cm, or nodules that enlarge over a time interval).

### Diagnosis

#### Diagnosis of lesions <1 cm

Pathology studies have shown that the majority of nodules smaller than 1 cm, which can be detected in a cirrhotic liver, are not HCCs. In these cases, a tighter follow-up with three-monthly US should be done. If the size does not change, surveillance every 3 months should be continued; if the diameter changes, the nodule should be diagnosed according to its size. After 2 years of this tighter follow-up, if there are no changes, the 6-month surveillance follow-up can be resumed.

#### Diagnosis of lesions ≥1 cm

If the diameter is ≥1 cm, the characteristic findings on dynamic CT/MR of arterial hyperenhancement with “washout” in the portal venous or delayed phase are highly specific and sensitive for a diagnosis of HCC. However, these criteria should not be used in patients with no baseline hepatic disease. On the other hand, a lesion that displays these findings on contrast US may also be a cholangiocarcinoma, making this technique less suitable for the noninvasive diagnosis of HCC. It is not useful for tumor staging either.

Several studies have shown that dynamic MRI has a slightly better performance than CT for the diagnosis of HCC, although there were limitations to these studies [7]. Therefore, one should utilize the locally available expertise, whether MRI or CT. In all cases, they should be performed using standardized technical specifications.

Alpha-fetoprotein should not be used as a diagnostic test due to the possibility of elevated levels in patients with non-HCC malignancies and nonmalignant diseases.

In those who do not have these characteristic features, a directed biopsy of the mass may be needed in order to confirm a diagnosis of HCC. However, there is no indication for biopsy of a focal lesion in a cirrhotic liver when the patient is a candidate for resection, or in patients with poor performance status or multiple comorbidities.

Pathological diagnostic criteria for HCC and the differential diagnosis with dysplastic lesions have been proposed [8]. Stromal invasion or tumor cell invasion into the portal tracts or fibrous septa defines HCC and is not present in dysplastic lesions.

### Recommendations

- Surveillance should be offered to patients with a high risk of developing HCC [1, B]
- An abdominal ultrasound (US) every 6 months is the method of choice [1, A]. Serum AFP is not suitable for screening purposes [II, B].
- Appropriate recall procedures should be in place in case a nodule is found in a screening US [2, D]
- Most lesions <1 cm in a cirrhotic liver will not be HCC, and they should be followed closely with three-monthly US [3, D]
- A radiologic diagnosis with multiphasic computed tomography (CT) or magnetic resonance (MRI) imaging is possible with cirrhotic patients if the findings of arterial hyperenhancement with “washout” in the portal venous or delayed phase are seen [2, D]
- A biopsy should be performed in case these criteria are not met, or there is no baseline hepatic disease [2, D]
- Alpha-fetoprotein should not be used as a diagnostic test [2, D].

### Staging

Both the extension of the diagnosis and the basal hepatic cellular injury determine the prognosis of hepatocellular carcinoma (HCC).

In addition to giving prognostic information, staging should allow guiding treatment options, defining their impact and facilitating the exchange of information in a standardized way [9].

**Fig. 1:**
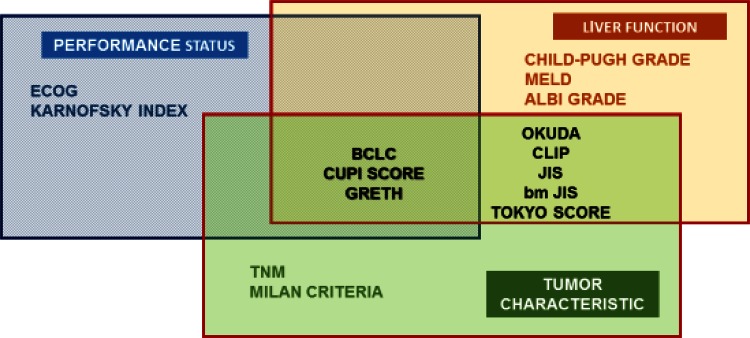
HCC staging systems and parameters. Modificadd de benyamad et al. (Clin Liver Dis 19 (2015): 277–294). *ECOG* Eastern Cooperative Oncology Group, *BCLC* Barcelona Clinic Liver Cancer, *CUPI SCORE* Chinese University Prognostic Index, *GRETCH* Groupe d’Etude et de Traitement du Carcinome Hepatocellulaire, *MELD* model for end-stage liver disease, *ALBI* albumin-bilirubin, *OKUDA* OKUDA staging system, *CLIP* Cancer of the Liver Italian Program, *JIS* Japanese integrated staging, *bm-JIS* biomarker-combined JIS, *TNM* tumor-node-metastasis staging

Systems of classification for the staging of HCC establish different scores based on clinical parameters related to the situation and tumor characteristics, liver functionality and the general state of health of the affected patient (Fig. 1). Stages are correlated in each set with the prognosis, and some classifications provide therapeutic guides with information on prognosis after application [10, 11].

**Fig. 2:**
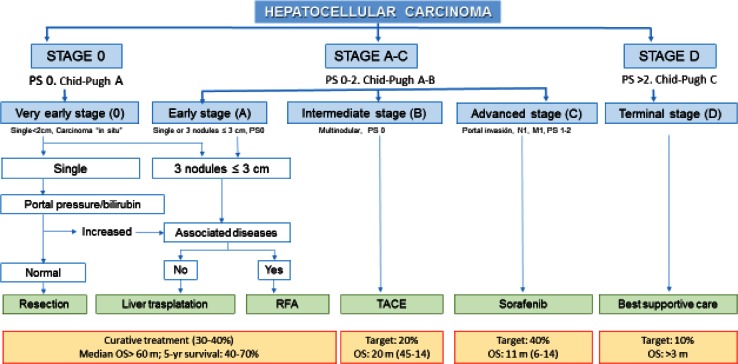
*BCLC* Barcelona Clinic Liver Cancer, *PS* performance status, *N* node classification, *M* metastasis classification, *RFA* radiofrequency ablation, *TACE* transcatheter arterial chemoembolization

There is no consensus as to which classification predicts better survival rates in patients with HCC. The classification of the Barcelona Clinic Liver Cancer Staging System (BCLC) has been validated externally and is endorsed by the European Association for the Study of the Liver (EASL) and by the American Association for the Study of Liver Disease (AASLD) [11]; this is standard procedure in occidental countries [12] (Fig. 2).

### Recommendation

The BCLC staging system has been validated externally, and it collects information on the situation of the tumor, liver functionality and the general condition of the patient; it also establishes therapeutic recommendations with prognostic information after treatment, and it is on the basis of these factors that we make our recommendation (level of evidence 2A; level of recommendation 1B level).

### Management of local disease: liver resection (LR) and liver transplantation (LT)

In general, LR is preferred in early-stage HCC patients who have no cirrhosis or well-preserved liver function, whereas LT is recommended for those patients with a compromised liver function.

LR should be offered to patients with solitary or limited multifocal HCC (stage BCLC-A), with no major vascular invasion or extrahepatic spread, no portal hypertension (defined as hepatic venous pressure gradient <11 mmHg or platelet count >100.000), adequate liver reserve (Child-Pugh class A and highly selected Child-Pugh class B7) and an anticipated liver remnant of at least 30–40 % in patients with cirrhosis and at least 20 % in noncirrhotic patients (evidence 2A; recommendation 1B) [13].

Anatomical resections are recommended (evidence 3A; recommendation 2C). Expected perioperative mortality rate of LR in cirrhotic patients is in the range of 2–3 %.

Adjuvant therapies after LR (e.g., sorafenib) have not been shown to improve outcome, and observation is the standard of care (evidence 1A; recommendation 1A) [14].

Assessment of the future liver remnant volume performed by CT or MRI volumetry can help to both predict post-LR liver function and select patients who may benefit from preoperative liver hypertrophy-inducing maneuvers.

Portal vein embolization (PVE) resulted in an increase of 8–27 % in future liver remnant volume with a morbidity rate of 2.2 % and no mortality (evidence 3A; recommendation 2C) [15]. Another hypertrophy-inducing strategy is the associating liver partition with portal vein ligation for staged hepatectomy (ALPPS) approach. However, this procedure is associated with a morbidity rate of 68 % and a mortality rate of 12 % [16].

The first randomized controlled trial to investigate whether LR (partial hepatectomy) or transcatheter arterial chemoembolization (TACE) yields better outcomes in patients with resectable multiple HCC, conducted on 173 Asian patients, found a survival advantage for LR over TACE (41 vs. 14 months) (evidence 2A; recommendation 2C) [17].

Patients within Milan criteria (MC) (single HCC nodule <5 cm or up to 3 nodules <3 cm each, with no macrovascular involvement and no extrahepatic disease) could be considered for LT (from either a dead or living donor) (evidence 2A; recommendation 1A), achieving a 5-year overall survival of more than 70 % and a 5-year recurrence rate of <10 % [18]. Perioperative mortality and 1-year mortality are expected to be approximately 3 % and <10 %, respectively.

Bridge or downstaging strategies could be considered in selected cases, if the waiting list for LT exceeds 6 months (evidence 2D; recommendation 2B). Nonetheless, in those cases exceeding MC, neoadjuvant treatments or “bridging therapies” to downstaging tumors to MC for LT are not recommended (evidence 2D; recommendation 2C) [19].

Patients with tumor characteristics slightly beyond MC and without microvascular invasion may be considered for LT. However, this indication requires prospective validation (evidence 2B; recommendation 2B). In the absence of molecular markers, both tumor size and number are important factors of post-LT recurrence that should be taken into account whenever selecting HCC patients beyond MC for LT.

### Management of local disease: local ablative treatment

Local ablation is considered the first-line treatment option for patients at early stages, not suitable for liver transplantation or surgery, or a therapeutic option avoiding tumor progression until liver transplantation (evidence 2A; recommendation 1B).

These therapies are based on the injection of substances in the tumor (ethanol, acetic acid), or on changes in temperature [radiofrequency ablation (RFA), microwave, laser, cryotherapy].

The most widely used are percutaneous ethanol injection (PEI) and RFA. Other ablative techniques such as microwave and cryoablation are still under investigation [20].

Both RFA and PEI have excellent results in tumors ≤2 cm (90–100 % complete necrosis), but for bigger tumors, the probability of achieving a complete necrosis is greater with RFA (evidence 1A: recommendation 1C). Five randomized controlled trials and two large meta-analyses showed that RFA obtains a better survival in early HCC, especially for tumors >2 cm [20, 21].

Currently, RFA stands as the best ablative treatment in tumors of <5 cm, but it has some limitations in cases where it is not technically feasible (tumors located close to other organs or large vessels). In these situations (10–15 %), PEI is recommended [20, 22] (evidence 1D; recommendation 1A).

The recurrence rate after percutaneous treatment is as high as for surgical resection, and it may achieve 80 % at 5 years [22].

### Management of locally advanced disease

The management of locally advanced disease includes transarterial chemoembolization (TACE), radioembolization and radiotherapy. These strategies can also be used in patients with early-stage HCC and with contraindications for radical therapies, and prior to liver transplants in patients who are estimated to have a long waiting time for their operation.

### Transarterial chemoembolization (TACE)

#### *Indications*

TACE is indicated for those patients with large or multifocal HCCs that are not amenable to resection or local ablation, with well-preserved hepatic function (i.e., Child-Pugh A or B cirrhosis), a good performance status and no vascular invasion, main portal vein thrombosis, extrahepatic disease spread, encephalopathy or biliary obstruction.

#### *Methodology*

TACE consists of the injection of a chemotherapeutic agent into the hepatic artery with or without lipiodol, and with or without a procoagulant material. TACE is currently available in some centers using drug-eluting beads (DEBs) [23].

#### *Efficacy*

TACE improves overall survival; rates of 2 years have been reported in randomized trials, around 31–63 %. TACE induced partial or complete response in 15–55 % of patients [24–26]. DEB-TACE induced similar rates of objective response and disease control compared with conventional TACE and has also been associated with improved tolerability with a significant reduction in serious liver toxicity and a significantly lower rate of doxorubicin-related side effects.

#### *Repeated TACE*

TACE should be limited to the minimum number of procedures necessary to control the tumor.

#### *Combination therapy*

The potential additive effect of combined therapy (sorafenib + TACE) over TACE alone has been directly addressed in two randomized phase II trials and a single randomized phase III trial, none of which suggest clear benefit [27, 28].

#### *Summary*

TACE recommendations: TACE is recommended for patients with asymptomatic large or multifocal HCC (BCLC stage B) with normal hepatic function and without vascular invasion or extrahepatic spread (evidence 2A; recommendation 1A).

### Radioembolization

Radioembolization using intraarterial injection of labeled microspheres induces extensive tumor necrosis (occluding small vessels combined with the emission of radiation in the tumor bed) with an acceptable safety profile.

#### *Indications*

It could be considered as an alternative to TACE for patients with advanced HCC who are candidates for TACE, but who have macrovascular invasion such as a branch or lobar portal vein thrombosis [29].

#### *Summary*

Radioembolization with Y-90 spheres is an alternative to TACE in cases of macrovascular invasion, excellent liver function and the absence of extrahepatic spread (evidence 3C; recommendation 3C).

### Radiotherapy

#### *Technique*

There are two approaches: intensity-modulated RT [IMRT] and image-guided stereotactic body radiotherapy [SBRT]).

#### *Indications*

3D-CRT is a reasonable option for patients who have failed other local modalities and have no extrahepatic disease, limited tumor burden and relatively well-preserved liver function. SBRT could also be recommended for patients with relatively small HCCs, who either are inoperable or refuse surgery and other local ablation techniques (evidence 3C; recommendation 3C).

#### *Combined therapy*

3D-CRT combined with TACE is under study [30].

### Recommendations

Locoregional therapy (transarterial chemoembolization [TACE], radioembolization and RT] is the preferred treatment approach for patients that are not amenable to surgery or liver transplantation.

The choice of nonsurgical treatment modality is empiric and influenced by local expertise and institutional practice. Few trials have directly compared any of the available therapies with one another, and there is little consensus as to when one modality should be chosen over another. Patients with disease spread outside the liver, and patients with major portal vein thrombosis should be considered for systemic therapy rather than liver-directed therapies.

### Treatment of metastatic disease

The standard treatment for patients with tumors invading the portal vein, having nodes or distant disease with an ECOG PS 1–2 and liver function Child-Pugh A, is the sorafenib (400 mg/12 h), an oral multikinase inhibitor whose clinical benefit has been tested in two different clinical trials: the SHARP trial (NCT00105443) [31] where 602 patients with advanced HCC were randomized to receive either sorafenib, 400 mg twice daily, or a placebo. Overall survival was significantly longer in the sorafenib group (10.7 vs. 7.9 months in the placebo group; HR 0.69; 95 % CI 0.55–0.87, *p* < .001). A similar trial was performed in Asian countries with 226 patients with similar design [32]: The median overall survival was 6.5 months for the sorafenib group versus 4.2 months for the placebo group (HR 0.68; 95 % CI 0.50–0.93, *p* = .014). The most common Sorafenib-related adverse events were hand-foot skin reaction and diarrhea.

The efficacy of sorafenib for patients with Child-Pugh class B or C liver function remains unclear. On the other hand, trials are also ongoing to evaluate the benefit of sorafenib combined with either TACE or chemotherapy [33].

HCC is resistant to chemotherapy. The drugs used (doxorubicin, cisplatin…) achieve response rates of 10 % with no impact on survival.

Ramucirumab is a recombinant human IgG1 monoclonal antibody against VEGFR-2 and avoids binding of ligands VEGF-A, VEGF-C and VEGF-D. In order to explore new options in second line, a phase-3 clinical trial REACH (NCT01140347) [34] was conducted where patients stage C or B refractory or not amenable to locoregional therapy that had previously received sorafenib were randomly assigned to receive intravenous ramucirumab (8 mg/kg) or a placebo every 2 weeks. The investigators concluded that the second-line treatment with ramucirumab did not significantly improve survival over the placebo in this setting of patients. However, subgroup analysis has suggested a potential benefit for those patients with alpha-FP values >400 ng/ml, which is currently being assessed in a new clinical trial, focusing on this specific setting.

Tivantinib and other c-met inhibitors (INC280, foretinib, MSC2156119 J and golvatinib) are currently being evaluated in second line after sorafenib [35, 36]; its benefit must be elucidated in further clinical trials.

In patients with ECOG PS of 3–4 and/or poor liver function (Child-Pugh C), cancer therapy is not indicated, and only palliative care is recommended.

### Monitoring

There is no evidence to guide the optimal post-treatment surveillance strategy in patients undergoing locoregional therapy for HCC. Recommendations are based on the consensus that earlier identification of disease recurrence may facilitate patient eligibility for investigational studies, or other forms of treatment.

Patients who undergo a complete resection are at risk from disease recurrence and second primary HCCs. Most patients who experience recurrence after resection have recurrent disease confined to the liver. The main goal of post-treatment surveillance is early identification of disease that might be amenable to subsequent local therapy. The determination of AFP is recommended [37], if it was initially elevated, every 3 months for 2 years and then every 6 months, and imaging (CT or MRI) every 3–6 months for 2 years and then every 6–12 months.

Re-evaluation according to the initial workup should be considered in the event of disease recurrence.

## Appendix

**Table:** Summary of recommendations on the diagnostic and therapeutic management of hepatocellular carcinoma

*Surveillance*	
Should be offered to patients with high risk of developing HCC	E: 1B, R: 1A/B
An abdominal ultrasound every 6 months is the method of choice	E: 2B, R: 1B
Appropriate recall procedures should be performed in case of a nodule detected in the screening US	E: 2D, R: 1A
Most lesions <1 cm in a cirrhotic liver will be benign and should be followed carefully every 3 months	E: 3D, R: 2B
*Diagnosis*	
Reliable diagnosis may be done by CT scan or MRI imaging in cirrhotic patients	E: 2D, R: 1A
Biopsy should be performed in the absence of radiologic criteria for HCC in cirrhotic patients or in patients without baseline hepatic disease	E: 2D, R: 2A
Alpha-fetoprotein level should not be used as a diagnostic test	E: 2D, R: 2A
*Staging*	
BCLC staging classification provides useful information including liver function, tumor extension, patient ECOG and prognosis, as well as the recommended treatment option. We recommend its use outside clinical trials	E: 2A, R: 1B
*Treatment*	
LR should be offered to patients with solitary or limited multifocal HCC (stage BCLC-A), with no major vascular invasion or extrahepatic spread, no portal hypertension (defined as hepatic venous pressure gradient <11 mmHg or platelet count >100.000), adequate liver reserve (Child-Pugh class A and highly selected Child-Pugh class B7) and an anticipated liver remnant of at least 30–40 % in patients with cirrhosis and at least 20 % in noncirrhotic patients	E: 2A, R: 1B
Anatomical resections are recommended	E: 3A, R: 2C
Adjuvant therapies after LR (e.g., sorafenib) have not proved to improve outcome, and observation is the standard of care	E: 1A, R: 1A
Patients within the Milan criteria could be considered for liver transplantation	E: 2A, R: 1A
Local ablation is the standard of choice for patients at early stages, not suitable for liver transplantation or surgery	E: 2A, R: 1B
TACE is indicated for those patients with large or multifocal HCCs that are not amenable to resection or local ablation, with well-preserved hepatic function (i.e., Child-Pugh A or B cirrhosis), a good performance status and no vascular invasion, main portal vein thrombosis, extrahepatic disease spread, encephalopathy or biliary obstruction	E: 2A, R: 1A
Sorafenib is the only approved systemic therapy for advanced stages of hepatocellular carcinoma. Overall survival is prolonged only in Child-Pugh A patients	E: 1A, R:1A
